# Prevalence of germline *TP53* mutation among early onset middle eastern breast cancer patients

**DOI:** 10.1186/s13053-021-00206-w

**Published:** 2021-12-14

**Authors:** Abdul Khalid Siraj, Tariq Masoodi, Rong Bu, Sandeep Kumar Parvathareddy, Kaleem Iqbal, Saud Azam, Maha Al-Rasheed, Dahish Ajarim, Asma Tulbah, Fouad Al-Dayel, Khawla Sami Al-Kuraya

**Affiliations:** 1 Human Cancer Genomic Research, King Faisal Specialist Hospital and Research Center, P.O. Box 3354, 11211 Riyadh, Saudi Arabia; 2 Department of Oncology, King Faisal Specialist Hospital and Research Centre, P.O. Box 3354, 11211 Riyadh, Saudi Arabia; 3 Department of Pathology, King Faisal Specialist Hospital and Research Centre, P.O. Box 3354, 11211 Riyadh, Saudi Arabia; 4 Research Center, Human Cancer Genomic Research, King Faisal Specialist Hospital and Research Center, MBC#98-16, P.O. Box 3354, 11211 Riyadh, Saudi Arabia

**Keywords:** *TP53 *mutation, Breast cancer, Li-Fraumeni syndrome, Lifetime risk

## Abstract

**Background:**

The data on prevalence and clinical relevance of *TP53* germline mutations in early onset Middle-Eastern breast cancer (BC) is limited.

**Methods:**

We determined *TP53* germline mutations in a cohort of 464 early onset BC patients from Saudi Arabia using capture sequencing based next generation sequencing.

**Results:**

Germline *TP53* pathogenic mutations were found in 1.5% (7/464) of early onset Saudi BC patients. A total of six pathogenic missense mutations, one stop gain mutation and two variants of uncertain significance (VUS) were detected in our cohort. No *TP53* pathogenic mutations were detected among 463 healthy controls. *TP53* mutations carriers were significantly more likely to have bilateral breast cancer (*p* = 0.0008). At median follow-up of 41 months, *TP53* mutations were an unfavorable factor for overall survival in univariate analysis. All the patients carrying *TP53* mutations were negative for *BRCA1* and *BRCA2* mutations. Majority of patients (85.7%; 6/7) carrying *TP53* mutation had no family history suggestive of Li-Fraumeni Syndrome (LFS) or personal history of multiple LFS related tumors. Only one patient had a positive family history suggestive of LFS.

**Conclusions:**

*TP53* germline mutation screening detects a clinically meaningful risk of early onset BC from this ethnicity and should be considered in all early onset BC regardless of the family history of cancer, especially in young patients that are negative for *BRCA* mutations.

**Supplementary Information:**

The online version contains supplementary material available at 10.1186/s13053-021-00206-w.

## Background

*TP53* (RefSeq NM_000546.6) mutations with Li-Fraumeni syndrome (LFS) is an autosomal dominant inherited disease primarily associated with high-risk for wide variety of early onset neoplasms [1]. *TP53* gene mutations are present in up to 2-6% of breast cancer (BC) patients younger than 35 [2–5]. The National Cancer Institute reported a cumulative cancer incidence of 50% by age of 31 years among female carriers of *TP53* germline mutations [6]. Despite the establishment of known criteria to diagnose LFS [7], *TP53* mutation carriers have been reported in a large number of patients who have not fulfilled these criteria. De novo mutations in *TP53* are well-documented and the incidence could reach up to 20% [8]. Accessibility to next generation sequencing have helped in identifying *TP53* germline mutations in individuals who do not fulfill clinical criteria previously recommended for LFS testing [9, 10].

In Saudi Arabia, BC is the most common cancer affecting women and it accounts for about 30% of all cancers diagnosed in women [11]. Interestingly, median age of diagnosis of BC among Saudi women is 50 years [11, 12], which is 10 years younger than those from western population [13–15]. Therefore, exploring the inherited germline mutations in cancer predisposition genes such as *TP53* is of great importance in this population.

However, data on the frequency of *TP53* germline mutations in young Saudi BC patients is limited [16]. To address this issue, in the current study, we screened 464 young Saudi breast cancer patients for *TP53* germline mutation. We investigated the prevalence and spectrum of *TP53* mutations in the entire cohort regardless of the family history and investigated the clinico-pathological characteristics of *TP53* mutation carriers.

## Methods

### Patient samples and data collection

Four hundred and sixty-four patients with early-onset breast cancer diagnosed between 1990 and 2015 at King Faisal Specialist Hospital and Research Centre (KFSHRC) were included in the study. Patients presenting with only ductal carcinoma in-situ were not included. Detailed clinico-pathological data, including follow-up data, were noted from case records and have been summarized in Table 1. Family history was collected from case records or by telephonic interview. 2019 World Health Organization (WHO) classification of breast tumors was used to classify the histologic subtype of each breast tumor sample. Overall survival was defined as the length of time from the date of diagnosis, that patients diagnosed with the disease are still alive. As controls, we analyzed a cohort of 463 age and gender matched cancer-free individuals for whom exome sequencing data was available in local population database. All the individuals of the control cohort were of the same ethnicity. Institutional Review Board of KFSHRC provided ethical approval for the current study. Research Advisory Council (RAC) granted waiver of informed consent for use of retrospective patient case data under project RAC# 2140 008. The patient samples were de-identified by assigning a unique number to each sample which could not be traced back to the individual patient. All the methods were carried out in accordance with relevant guidelines and regulations.

**Table 1:** Clinico-pathological variables for the patient cohort (*n*=464)

Clinico-pathologic variables	n (%)
**Age (years)**	
≤30	77 (16.6)
31 - 40	387 (83.4
Median (in years)	36.0
Range(IQR)^	32.0 – 39.0
**Family history of breast cancer**	
Yes	55 (11.9)
No	409 (88.1)
**Family history of any cancer**	
Yes	91 (19.6)
No	373 (80.4)
**Personal history of other cancer**	
Yes	5 (1.1)
No	459 (98.9)
**Bilateral breast cancer**	
Yes	4 (0.9)
No	460 (99.1)
**Histological type**	
Infiltrating Ductal carcinoma	421 (90.7)
Infiltrating Lobular carcinoma	19 (4.1)
Mucinous carcinoma	10 (2.2)
Others	14 (3.0)
**Tumor size**	
≤2 cm	137 (29.5)
>2 cm	311 (67.1)
Unknown	16 (3.4)
**Lymph node status**	
Negative	164 (35.4)
Positive	284 (61.2)
Unknown	16 (3.4)
**Distant metastasis**	
Absent	411 (88.6)
Present	37 (8.0)
Unknown	16 (3.4)
**Histologic Stage**	
I	71 (15.4)
II	170 (36.6)
III	170 (36.6)
IV	37 (8.0)
Unknown	16 (3.4)
**Histologic Grade**	
Well differentiated	29 (6.3)
Moderately differentiated	190 (40.9)
Poorly differentiated	218 (47.0)
Unknown	27 (5.8)
**Estrogen Receptor**	
Positive	268 (57.7)
Negative	196 (42.3)
**Progesterone Receptor**	
Positive	238 (51.3)
Negative	226 (48.7)
**Her-2 neu**	
Positive	176 (37.9)
Negative	288 (62.1)
**Molecular subtype**	
Luminal	303 (65.3)
Her-2 positive	73 (15.7)
Triple negative	88 (19.0)
***BRCA1*mutation**	
Present	41 (8.8)
Absent	423 (91.2)
***BRCA2*mutation**	
Present	16 (3.4)
Absent	448 (96.6)
**Survival Duration (in months)**	
Median	55.1
Range(IQR)^	27.0 – 79.0

^ IQR, inter quartile range

### DNA isolation

DNA samples were extracted from formalin-fixed and paraffin-embedded non-tumor tissues utilizing Gentra DNA Isolation Kit (Gentra, Minneapolis, MN, USA) according to the manufacturer’s protocols as elaborated in the previous studies [17]. The non-tumor tissues were selected from normal tissues adjacent to the tumor tissue or from normal tissues from other organ sites operated for an unrelated disease. The normal tissues were confirmed by histopathological examination.

### Targeted capture sequencing and mutation calling

Targeted capture sequencing was performed on 464 breast cancer samples using Illumina platform. Pre-alignment quality metrics were obtained using FastQC (http://www.bioinformatics.babraham.ac.uk/projects/fastqc/) and quality passed sequencing reads were aligned to the human reference genome hg19 using Burrows-Wheeler Aligner (BWA) [18]. Local realignment was performed and PCR duplicates were marked using Picard tools (http://broadinstitute.github.io/picard/). In order to obtain high quality mutation calls, base-quality recalibration and variant calling was performed with GATK [19]. Post alignment quality metrics were obtained using GATK. The identified variants were annotated using ANNOVAR [20]. *TP53, BRCA1 and BRCA2* variants with a minor allele frequency of greater than 0.001 as found in dbSNP, the National Heart, Lung, and Blood Institute exome sequencing project, 1000 Genomes and Exome Aggregation Consortium (ExAC) were excluded for further analysis. A variant was considered a true positive if the variant allele frequency (VAF) was at least 20% with sequencing depth in the variant location region to be >=20. All the mutations were also manually checked using the Integrated Genomics Viewer (IGV) to filter out artifacts. The control group included 463 cancer-free women age less than 40 years for whom whole exome sequencing (WES) data was available. *TP53* mutations were extracted from WES and similar filters and pathogenicity classification was applied.

### Pathogenicity of variants

All the variants were classified according to The American College of Medical Genetics and Genomics (*ACMG*) guidelines for *TP53* gene [21]. Further, variants were also scored for likelihood of pathogenicity using Combined Annotation Dependent Depletion (CADD) [22], Align GVGD [23] and BayesDel [24]. VUSs according to ACMG were considered likely pathogenic if predicted pathogenic by two of the three prediction tools.

### Mutation validation by PCR and sanger sequencing

To validate the mutations identified by Capture sequencing technology, Primer 3 software was used to design the primers for each mutation (available upon request). PCR was performed in a total volume of 25 µl with 20 ng of genomic DNA, 2.5 µl 10 x Taq buffer, 2.3 mM dNTPs, 1 unit Taq polymerase and 0.2 µM each primer and de-ionized water. The efficiency and quality of the amplified PCR products was confirmed by loading them on a 2% agarose gel.

For Sanger sequencing, the PCR products were subsequently subjected to direct sequencing with BigDye terminator V 3.1 cycle sequencing reagents and analyzed on an ABI 3730XL DNA analyzer (Applied Biosystems, Foster City, CA). Reference sequences were downloaded from NCBI GenBank. Sequencing traces were analyzed with the Mutation Surveyor v4.04 (Soft Genetics, LLC, State College, PA).

### Tissue microarray (TMA) construction and immunohistochemistry (IHC) staining

TMA construction was performed as described earlier [25]. Briefly, tissue cylinders with a diameter of 0.6 mm were punched from representative tumor regions of each donor tissue block and brought into recipient paraffin block using a modified semiautomatic robotic precision instrument (Beecher Instruments, Woodland, WI). Two cores of breast cancer were arrayed from each case.

IHC staining was performed manually with staining and scoring of estrogen receptor (ER), progesterone receptor (PR) and Her-2 neu performed as described previously [26]. Briefly, the cutoff for ER and PR was taken as 1% nuclear staining, whereas HER2 overexpression was assessed according to American Society of Clinical Oncology/College of American Pathologists (ASCO/CAP) guidelines [27].

### Statistical analysis

The associations between clinico-pathological variables and *TP53* mutation was performed using contingency table analysis and Fisher exact test. Mantel-Cox log-rank test was used to evaluate overall survival. Survival curves were generated using the Kaplan-Meier method. Cox proportional hazards regression model was used for multivariate analysis. Two-sided tests were used for statistical analyses with a limit of significance defined as p value < 0.05. Data analysis was performed using the JMP14.0 (SAS Institute, Inc., Cary, NC) software package.

## Results

### *TP53* mutations and their clinico-pathological characteristics

In our cohort, a total of nine mutations were identified in early onset Saudi BC patients by Capture sequencing and further validated by Sanger sequencing technology. Seven of these mutations were found pathogenic/likely pathogenic (1.5%, 7/464) and other two as variants of uncertain significance (VUS) by ACMG guidelines for *TP53* gene [21]. One VUS was predicted pathogenic by all three prediction tools whereas other was predicted pathogenic by only CADD. Most of the mutations observed in *TP53* gene were missense (six mutations) along with one stop gain mutation (Table 2). However, none of the pathogenic mutations in *TP53, BRCA1* and *BRCA2* were detected among 463 matched healthy controls. Due to the limitation of Capture sequencing technology, large mutations (>300 – 400 base pairs) cannot be identified.

**Table 2:** Mutation classification according to ACMG TP53* guidelines and computational predictions

Chr	Position	Ref	Alt	Amino Acid	Type	CADD	aGVGD	BayesDel	ACMG_*TP53*	Alt Depth	Total Depth	VAF
17	7,579,899	T	A	p.Q5L	Missense	6.003	Class C0	0.197777	Likely Pathogenic	40	94	42.6
17	7,577,121	G	A	p.R273C	Missense	25.5	Class C65	0.433271	Likely Pathogenic	256	510	50.2
17	7,577,094	G	A	p.R282W	Missense	26	Class C65	0.542691	Likely Pathogenic	693	1400	49.5
17	7,577,022	G	A	p.R306X	Stop gain	37	NA	0.625005	Pathogenic	186	549	33.9
17	7,573,988	C	T	p.A347T	Missense	27.2	Class C0	0.152476	VUS	195	421	46.3
17	7,578,508	C	T	p.C141Y	Missense	23.8	Class C65	0.561428	Likely Pathogenic	917	2904	31.6
17	7,577,548	C	T	p.G245S	Missense	28.9	Class C55	0.550935	Pathogenic	202	464	43.5
17	7,577,538	C	T	p.R248Q	Missense	28.6	Class C35	0.377622	Pathogenic	170	193	88.1
17	7,577,093	C	A	p.R282L	Missense	27.5	Class C65	0.416469	VUS	56	125	44.8

*TP53 RefSeq NM_000546.6

Note: For aGVGD, Class C15 and higher are considered pathogenic; for BayesDel, scores ≥ 0.16 are considered pathogenic; for CADD, scores ≥ 20 are considered pathogenic.

Median age of the *TP53* mutant cases was 32 years (range: 22 – 39 years) at the time of diagnosis. Of the patients harboring *TP53* mutation, 2 (28.6%) patients underwent modified radical mastectomy, 4 (57.1%) had simple mastectomy and 1 (14.3%) had lumpectomy. All the seven tumors were of infiltrating ductal carcinoma histologic subtype. Two (28.6%) patients presented with grade 2 tumor, whereas five (71.4%) patients had grade 3 tumors. Lymph node metastasis was noted in two (28.6%) patients and distant metastasis was present in three (42.9%) patients. Two (28.6%) patients presented with stage II tumor, two (28.6%) with stage III and three (42.9%) with stage IV tumors. Bilateral breast cancer was present in two (28.6%) patients. One (14.3%) patient had triple negative breast cancer. Family history was positive in one (14.3%) patient; with malignancies noted in five first degree relatives (rhabdomyosarcoma, cerebellar astrocytoma, osteosarcoma, oligodendroglioma and pancreatic cancer). Three (42.9%) patients received neoadjuvant chemotherapy and all the patients received adjuvant chemotherapy. Four (57.1%) patients received radiotherapy. All the seven cases were negative for *BRCA1/2* mutations (Supplementary table S1).

Median follow-up for the seven patients was 41 months (range: 25 – 50 months). During the follow-up, one patient developed local recurrence as well as liver metastasis and died due to disease progression (survival = 50 months). Another patient died after 25 months of follow-up due to disease progression, with metastasis involving the brain. The remaining five patients were alive at the time of last follow-up (Supplementary table S1).

### Clinico-pathological associations of *TP53* mutation carriers

**Fig. 1:**
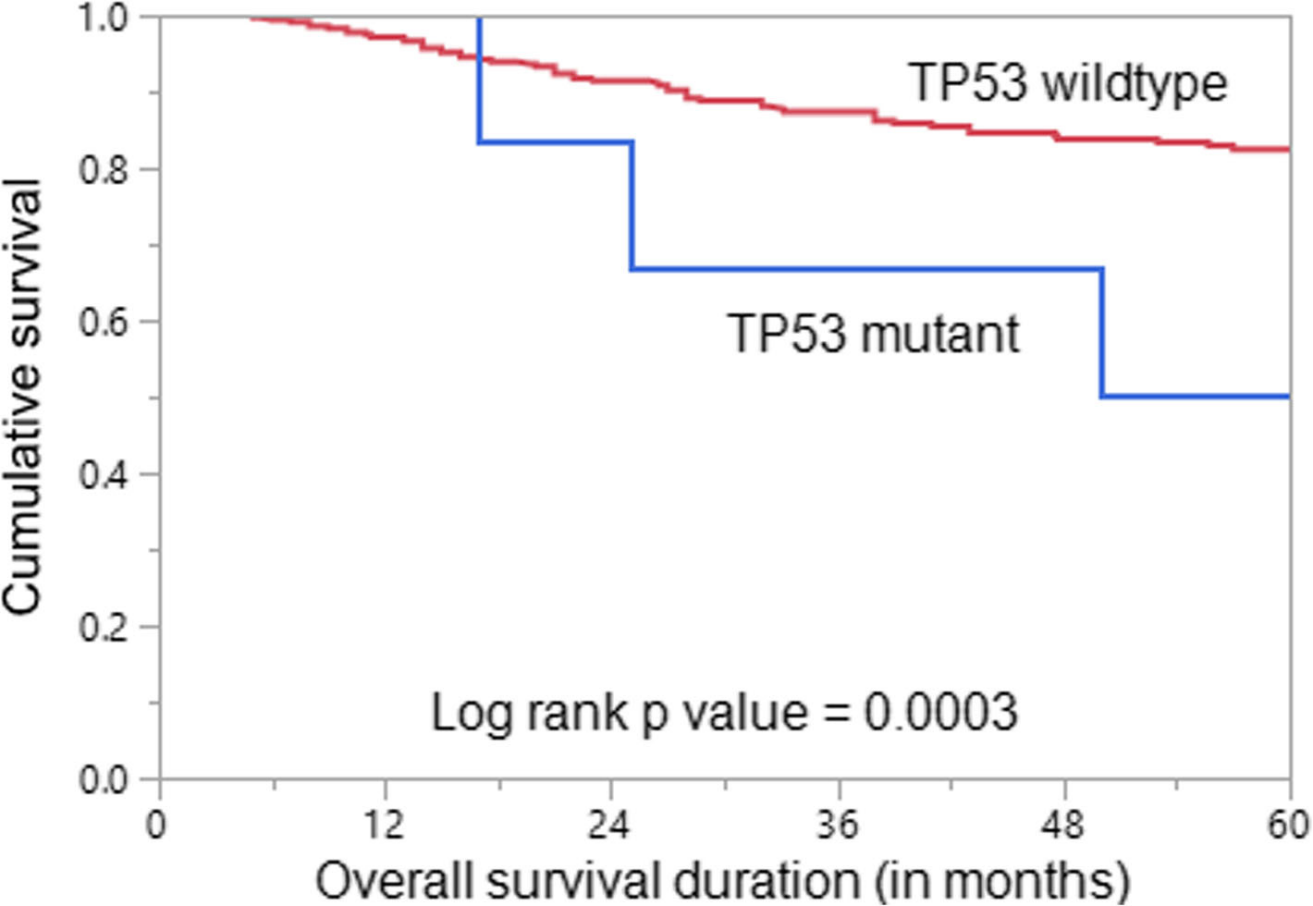
Survival Analysis of *TP53* mutation in breast cancer. Kaplan Meier survival plot showing statistically significant poor overall survival in *TP53* mutant cases compared to *TP53* wild-type cases (*p* = 0.0360)

We analyzed the association between *TP53* mutation and clinico-pathological characteristics among 407 BC patients (after excluding BRCA mutant cases). We found a significant association between *TP53* mutation and patients with bilateral breast cancer (*p* = 0.0008) as well as distant metastasis (*p* = 0.0139). Importantly, *TP53* mutations were associated with poor overall survival (*p* = 0.0003) (Table 3; Fig. 1). However, on multivariate analysis, *TP53* mutations were not an independent predictor of overall survival.

**Table 3:** Summary of clinico-pathological variables in *TP53* mutant breast cancer patients age ≤ 40 years, after excluding *BRCA* carriers (*n* = 407)

Clinico-pathological variables	*TP53* carriers (*n* = 7)	*TP53* non-carriers (*n* = 400)	p value
	**n (%)**	**n (%)**	
**Age at diagnosis, years**			
Mean ± SD	32.0 ± 6.0	34.9 ± 4.6	0.2410
Median (range)	32 (22 – 39)	36 (13 – 40)	0.1380
≤30	3 (4.7)	61 (95.3)	0.0813
31 – 40	4 (1.2)	339 (98.8)	
**Family history of breast cancer**			
Yes	0 (0.0)	42 (100.0)	1.0000
No	7 (1.9)	358 (98.1)	
**Family history of any cancer**			
Yes	1 (1.4)	71 (98.6)	1.0000
No	6 (1.8)	329 (98.2)	
**Personal history of other cancer**			
Yes	0 (0.0)	4 (100.0)	1.0000
No	7 (1.7)	396 (98.3)	
**Bilateral breast cancer**			
Yes	2 (66.7)	1 (33.3)	0.0008*
No	5 (1.2)	399 (98.8)	
**Tumor size**			
≤2 cm	1 (0.8)	120 (99.2)	0.4441
>2 cm	6 (2.2)	264 (97.8)	
**Lymph node status**			
Negative	3 (2.2)	134 (97.8)	0.6996
Positive	4 (1.6)	250 (98.4)	
**Distant metastasis**			
Absent	4 (1.1)	355 (98.9)	0.0139*
Present	3 (9.4)	29 (90.6)	
**Stage**			
I	0 (0.0)	63 (100.0)	0.0481*
II	2 (1.4)	144 (98.6)	
III	2 (1.3)	148 (98.7)	
IV	3 (9.4)	29 (90.6)	
**Histologic Grade**			
Well differentiated	0 (0.0)	28 (100.0)	0.3018
Moderately differentiated	2 (1.1)	174 (98.9)	
Poorly differentiated	5 (2.8)	173 (97.2)	
**Estrogen receptor status**			
Positive	4 (1.6)	246 (98.4)	1.0000
Negative	3 (1.9)	154 (98.1)	
**Progesterone receptor status**			
Positive	3 (1.4)	217 (98.6)	0.7078
Negative	4 (2.1)	183 (97.9)	
**Her-2 neu status**			
Positive	3 (1.7)	169 (98.3)	1.0000
Negative	4 (1.7)	231 (98.3)	
**Molecular Subtype**			
Luminal	4 (1.4)	274 (98.6)	0.7464
Her-2 positive	2 (2.9)	68 (97.1)	
TNBC	1 (1.7)	58 (98.3)	
**Overall survival (5-years)**	33.3	82.5	0.0003*

*, significant p value

## Discussion

The prevalence of *TP53* mutations among women with early breast cancer has been explored in different populations [2, 28–31]. The accessibility to gene panel testing and next generation sequencing, in addition to the updated international guidelines which downplay the importance of positive family history of LFS [32, 33], have led to dramatic increase in *TP53* testing, especially among young BC patients. Therefore, we conducted this study to determine the *TP53* germline mutation in a large cohort of 464 Saudi women diagnosed with BC <40 years of age.

We found germline *TP53* mutation in 1.5% (7/464) of Saudi early-onset BC patients regardless the family history of cancer or personal history of multiple LFS-related tumors. None of these seven  mutations appeared to be recurrent. All detected mutations were missense mutations except one, which was stop gain. No pathogenic mutations were found in the control cohort.

The mutation rate in our study of 1.5% is lower than what other studies have reported where *TP53* mutation rate ranges from 3 to 8% in very early onset BC [2, 4, 28]. Although, in our cohort we used the cut-off age of early onset BC <40 years, decreasing the cut-off age to very early onset of BC <30 years did not show enrichment of *TP53* mutation as shown by others [4, 34, 35]. This could probably be due to ethnic differences in the prevalence among different population.

*TP53* mutation carriers had a significantly worse overall survival than non-carriers. However, in multivariate analysis, this association was lost, which could partially be attributed to the small number of *TP53* mutation carriers in this cohort.

In our previous study including same group of samples, we determined the frequencies of the most common inherited germline mutations, *BRCA1* and *BRCA2*. Although we found *BRCA1* prevalence of 8.8% and *BRCA2* prevalence of 3.4% in the entire cohort, none of the *TP53* germline mutation carriers were positive for *BRCA1* or *BRCA2* mutations. This is consistent with previous reports where *TP53* mutations are seen in early-onset BC patients that are negative for *BRCA1/2* pathogenic variants [30, 31, 36].

*TP53* mutations carriers were more likely to have bilateral BC compared to non-carriers in our study. However, we did not find *TP53* mutations to be associated with HER2 positive cancers. Several previous studies have shown that women carrying germline *TP53* mutations were diagnosed with HER2 positive tumors [29, 37–39]. Among our mutation carriers, only three out of seven (42.9%) had HER2 (+3) receptor expression. Which indicates that HER2 amplification in Saudi population might not be a useful marker in identifying *TP53* mutations. Recent large study conducted on Chinese population couldn’t identify the association between *TP53* mutations and HER2 positivity in BC patients [34]. Whether the lack of association observed in our study is due to sample size or true reflection of ethnic difference in BC need to be further evaluated through additional studies.

An intriguing finding is that most of the *TP53* mutations carriers have negative family or personal history of cancer. Only one patient met the criteria of LFS or LFL syndrome. This is of important clinical implications, given the socio-cultural barriers to accurately documenting family history of cancer and lack of early BC awareness make genetic testing of *TP53* in young BC patient an important strategy to identify BC patient with hereditary BC.

Overall, our study has shown the spectrum of *TP53* germline mutation in Saudi cohort. The differences in frequency of *TP53* mutation, and clinical characteristics such as lack of *TP53* enrichment at very early onset (≤ 30 years of age) BC and the lack of association with HER2 status further suggest that *TP53* carriers may vary across different ethnicities and countries. We therefore propose that women with breast cancer before the age of 40 to be screened for *TP53* mutations even with no family history of cancer.

Understanding of *TP53* mutation prevalence coupled with screening for these selected women will not only be beneficial for patients but also for their families by adopting specific surveillance options for early cancer detection and/or prevention. Furthermore, knowledge about *TP53* mutation may aid clinician to the best treatment modalities for these patient such as bilateral mastectomy to reduce the risk of a second primary breast cancer and minimizing the radiotherapy if possible since radiation therapy may increase risks in these patients [40, 41].

Despite the relatively large sample size of early onset BC, this study has certain limitations. Firstly, this is a retrospective and a single tertiary care center study, so selection bias cannot be ignored. Secondly, the low power in statistical analyses performed due to small number of mutant positive cases should be considered cautiously when interpreting the results. Thirdly, the socio-cultural barriers in this population may preclude accurate documentation of family history.

## Conclusions

In conclusion, *TP53* germline mutation screening detects a clinically meaningful risk of early onset BC from this ethnicity and should be considered in all early onset BC regardless of the family history of cancer, especially in young patients that are negative for *BRCA* mutations.

## Supplementary information

**Additional file 1**

**Additional file 2**

## Data Availability

Not applicable.
