# Supplementary figures and images for: Prevalence of germline TP53 mutation among early onset middle eastern breast cancer patients
Source: Hered Cancer Clin Pract. 2021 Dec 14;19:49. doi: 10.1186/s13053-021-00206-w (PMC8670057; doi:10.1186/s13053-021-00206-w)

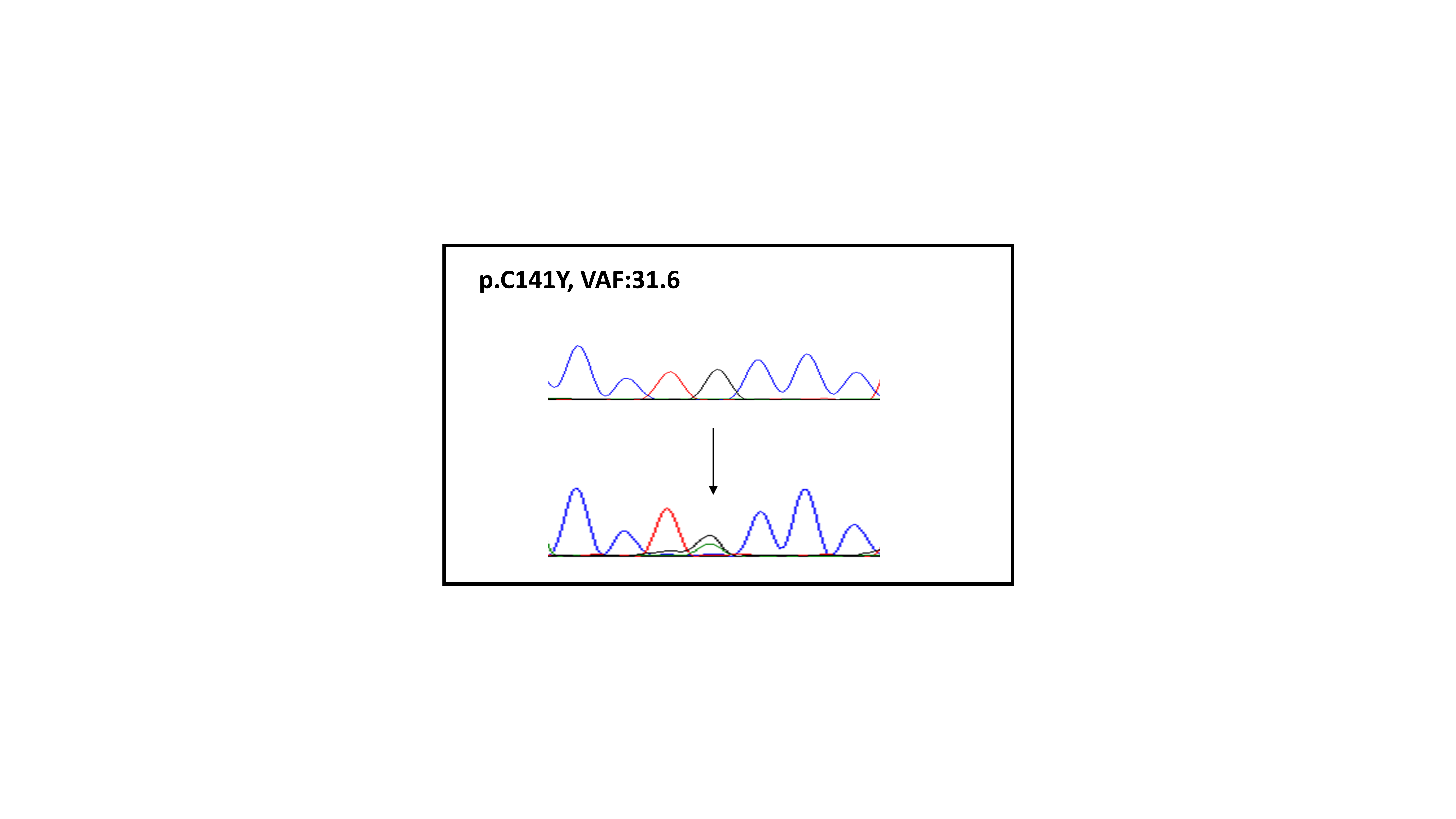

Supplement: Supplementary file 2 — Additional file 2 [file 13053_2021_206_MOESM2_ESM.tif]
